# Life History of *Rhamphorhynchus* Inferred from Bone Histology and the Diversity of Pterosaurian Growth Strategies

**DOI:** 10.1371/journal.pone.0031392

**Published:** 2012-02-15

**Authors:** Edina Prondvai, Koen Stein, Attila Ősi, Martin P. Sander

**Affiliations:** 1 Hungarian Academy of Sciences – Eötvös Loránd University “Lendület” Dinosaur Research Group, Eötvös Loránd University, Budapest, Hungary; 2 Steinmann Institut für Geologie, Mineralogie und Paläontologie, University of Bonn, Bonn, Germany; University of Maryland, United States of America

## Abstract

**Background:**

*Rhamphorhynchus* from the Solnhofen Limestones is the most prevalent long tailed pterosaur with a debated life history. Whereas morphological studies suggested a slow crocodile-like growth strategy and superprecocial volant hatchlings, the only histological study hitherto conducted on *Rhamphorhynchus* concluded a relatively high growth rate for the genus. These controversial conclusions can be tested by a bone histological survey of an ontogenetic series of *Rhamphorhynchus*.

**Methodology/Principal Findings:**

Our results suggest that Bennett's second size category does not reflect real ontogenetic stage. Significant body size differences of histologically as well as morphologically adult specimens suggest developmental plasticity. Contrasting the ‘superprecocial hatchling’ hypothesis, the dominance of fibrolamellar bone in early juveniles implies that hatchlings sustained high growth rate, however only up to the attainment of 30–50% and 7–20% of adult wingspan and body mass, respectively. The early fast growth phase was followed by a prolonged, slow-growth phase indicated by parallel-fibred bone deposition and lines of arrested growth in the cortex, a transition which has also been observed in *Pterodaustro*. An external fundamental system is absent in all investigated specimens, but due to the restricted sample size, neither determinate nor indeterminate growth could be confirmed in *Rhamphorhynchus*.

**Conclusions/Significance:**

The initial rapid growth phase early in *Rhamphorhynchus* ontogeny supports the non-volant nature of its hatchlings, and refutes the widely accepted ‘superprecocial hatchling’ hypothesis. We suggest the onset of powered flight, and not of reproduction as the cause of the transition from the fast growth phase to a prolonged slower growth phase. Rapidly growing early juveniles may have been attended by their parents, or could have been independent precocial, but non-volant arboreal creatures until attaining a certain somatic maturity to get airborne. This study adds to the understanding on the diversity of pterosaurian growth strategies.

## Introduction

The most commonly found pterosaur remains in the Solnhofen Limestones belong to the well-known genus *Rhamphorhynchus*, among which there is a striking variability in body size. Originally, based on size categories and other characters such as fusion state in different parts of the skeleton, cranial morphology, shape of the tail vanes and proportions of limb elements, Wellnhofer [1], [2] described five different *Rhamphorhynchus* species which can be listed in order of size increase as follows: *R. longicaudus*, *R. intermedius*, *R. muensteri*, *R. gemmingi*, *R. longiceps*. Two decades later, using statistical and morphological analysis, Bennett [3] concluded that all known specimens of *Rhamphorhynchus* belong to the same species *Rhamphorhynchus muensteri*. He suggested that the observed variability in size, fusion state and proportions can be attributed to the different ontogenetic stages represented by these specimens. Bennett [3] argued that the general prevalence of immature individuals among the vertebrate fossils from the Solnhofen Limestone [4], [5] also supports this view. He found two or more statistically distinguishable, discrete size-classes of *Rhamphorhynchus*, and defined them as year-classes reflecting seasonal mortality events. The first, second and third size categories included the small, medium and large specimens, respectively, which then had been referred to as juveniles, subadults and adults implying their ontogenetic status as well. With the introduction of year-classes Bennett [3] also indicated that, unlike most derived pterodactyloids [6]–[9], *Rhamphorhynchus* must have had slow, crocodile-like indeterminate growth with at least three years of growth after they had first become airborne. He suggested furthermore that, based on the wing proportions, all known specimens including the smallest ones could fly. He raised two alternative hypotheses to explain the suggested early onset of flight: either the hatchlings needed parental care until they reached the minimum size found in the fossil record to fly off, or they were precocial, being capable of flight shortly after hatching. The latter has been widely accepted [10]–[12], and further discussed by Unwin [9], who extended the hypothesis of early flight abilities to pterosaurs in general and suggested that they were superprecocial and able to fly immediately after hatching.

On the other hand, the only bone histological study ever conducted on *Rhamphorhynchus* [13] and studies of other, more basal and earlier pterosaur genera, such as the Late Triassic MGUH VP 3393 ([13], described first as *Eudimorphodon cromptonellus* [14]; later revised and considered a *nomen dubium* [15]), Early Jurassic *Dorygnathus* [16], *Dimorphodon* [7] and Middle Jurassic *Rhamphocephalus* [17], indicated growth rates that invariably exceeded that of extant crocodiles and other, non-avian sauropsids [9]. Although the bone microstructure found in the two investigated *Rhamphorhynchus* specimens implied relatively slower growth than in other and more basal pterosaurs, the growth strategy of this genus still seemed to resemble that of smaller extant birds rather than non-ornithodiran sauropsids [13]. The presence of fibrolamellar bone, that made up the bulk of the cortex in the smaller specimen, but has been preserved only in some patches in the larger one [13], refers to fast bone deposition at least during early ontogeny, and suggests that growth did not protract over several years [7]. Thus, Ricqlès et al. [7] presumed that, similarly to dinosaurs, high growth rates characterized pterosaurs in general, and that this feature was already shared with the common ornithodiran ancestor.

Based on the energetic trade-offs operating in all life history strategies [18], [19], it is unlikely that the predictions of these different approaches are simultaneously realized, namely that *Rhamphorhynchus* hatchlings were able to maintain fast growth while relying mainly on their powered-flight capabilities to take care of themselves without any parental help. Powered flight is one of the most energy-consuming locomotion types in tetrapods, therefore high growth rates and a superprecocial onset of the flying lifestyle in a highly developed hatchling are mutually exclusive developmental parameters [20]–[24]. The validity of this simple trade-off model is supported by the fact that the only extant superprecocial fliers, the megapod birds have very low if not the lowest growth rates among extant birds [20], [25], [26]. The same has been suggested for some supposedly superprecocial early birds like enantiornithines with slow growing lamellar-zonal bone tissue [27], [28], and might have applied to the basalmost birds *Archaeopteryx* and *Jeholornis* as well, the bone histology of which also refers to lower growth rate when compared to extant birds [29]. Hence, further investigation is needed to test which hypothesis is more plausible in the case of *Rhamphorhynchus*.

Currently, histological study of more specimens representing an ontogenetic series is the best approach to reveal the growth strategy of an extinct animal, and to extrapolate the results to its possible life history strategies. Until now the bone microstructure of only two specimens of *Rhamphorhynchus* belonging to the first and second size-classes of Bennett [3] has been investigated [13]. Clearly, an ontogenetic series should contain more specimens of diverse size-ranges, but *Rhamphorhynchus* has generally been considered too valuable for the destructive sampling procedure that is necessary for histological investigation. In spite of this, for the current study five *Rhamphorhynchus* specimens have been provided for histological sampling. Their relative body sizes and morphological properties indicate that these specimens represent different ontogenetic stages. Sampling specimens of an ontogenetic series is especially important in pterosaurs, because in advanced ontogenetic stages the thin-walled bones retain only a very incomplete record of their actual growth dynamics. The incompleteness of the growth record is due to the extensive medullary cavity expansion in which osteoclastic activity resorbs the primary bone along with any LAGs. This is followed by the activation of the endosteum which deposits secondary endosteal bone centripetally. This process is necessary in order to maintain the extremely low corticomedullary index [7] characteristic of pterosaurian bones. The current study thus represents the first histological investigation of an ontogenetic series of the genus *Rhamphorhynchus* whereby the competing hypotheses highlighted above are tested.

## Materials and Methods

Five specimens of *Rhamphorhynchus* were sampled for this study which can be ordered by increasing femoral length as follows: BSPG 1960 I 470a, BSPG 1877 X I, IPB 179, MTM V 2008.33.1., BSPG 1929 I 69. CM 11433 and RAM V97017/258, the only *Rhamphorhynchus* specimens hitherto subjected to histological investigation were used for comparative purposes in the recent evaluation. Measurements of further specimens were taken for estimating body sizes of incomplete studied specimens. Information on institutional abbreviations appearing in the inventor numbers of the concerned specimens is available in the supporting material (Text S1).

### Histological assessment

Considering the value of the specimens, only minute pieces from fracture surfaces of the diaphyseal cortex of BSPG and of most elements of MTM specimens were taken. Entire cross sections were taken from the femur and tibia of the IPB specimen using histological coring method [30] and from the tibia-fibula and the III. phalanx of the fourth manual digit (termed as the wingfinger) of MTM by sawing off the items transversally. The sampled bones included mainly hind limb elements, but also wing bones and a prepubis. Samples were thin-sectioned into transverse sections using standard techniques [e.g. 31]. Histological features of the selected bones were studied under a Leica DMLP polarized light microscope. Images were acquired with a Leica DFC420 digital camera and processed with Imagic ImageAccess software. Percentage of porosity (Pp%) was given for each specimen by calculating the proportion of the area of vascular spaces relative to the total area of the bone section from digital photos. This measure is intended to illustrate the variability of vascular degree among bones of the same specimen and the ontogenetic change in the porosity of bones. Where there were distinct superimposed layers of different tissue types, the porosity of each layer relative to its own area (Pp_l_%) as well as to the total vascular area of the section (Pp_t_%) were indicated to reveal the pattern of vascularization in the entire cortex. Area calculations were performed with AutoCAD 2010. More details about the specimens and the sampled bones are given in Table 1. Abbreviations standing for histological structures in the figures are summarized in the supporting material (Text S2).

**Table 1:** Tabulation of specimens studied, selected elements, femoral length, and some particulars of specimens.

Specimen	Sampled bone	Femoral length (mm)	Notes
BSPG 1960 I 470a	left tibia	10.2	smallest specimen, partial, articulated skeleton, compressed
BSPG 1877 X I	left femur	12.6	almost complete, articulated skeleton without skull, compressed
BSPG 1929 I 69	right femur, left tibia	51.4	largest specimen, partially articulated, almost complete skeleton, compressed
IPB 179	right femur, right tibia	24.8	articulated hindlimb, well preserved
MTM V 2008.33.1	left ulna, III phalanx of right wingfinger, left femur, right tibia, left prepubis	30	partially articulated, complete skeleton, well preserved

### Size-classes vs. ontogenetic stage

One of the aims of the study was to use histological features of *Rhamphorhynchus* to test whether Bennett's [3] three size-classes indeed reflect real ontogenetic ages (juvenile, subadult, adult). Each investigated specimen was assigned to one of Bennett's size categories. In order to reveal the relationship between overall histological pattern and size-classes, distinct ontogenetic stages were established based on histological characters. Hatchlings and early juveniles form the first group and are characterized by intensively growing bone tissues with high degree of porosity. The next ontogenetic category includes late juveniles and subadults the bone microstructure of which suggests a considerable decrease in bone growth rate relative to that found in the first group, but which still exhibits signs of active growth. The last group is composed of adults or skeletally mature individuals that reveal substantial decrease or complete cessation of growth in their bone histology. If Bennett's [3] size-classes are good indicators of relative ontogenetic stages of the classified specimens, then the histologically defined ontogenetic categories should fit more or less the corresponding size-classes. Estimating absolute ages, however, abounds in uncertainties and is beyond the scope of this paper.

#### Size estimates

Wingspan and body mass estimates were used as measures of size to correlate histological features of each specimen to its relative body size. Wingspan estimates were performed in CorelDRAW 12. We measured the length of all wing elements from the humerus to the last phalanx of the wingfinger from digital photos, and substituted these wing elements with line-objects of corresponding length. Using a digitized skeletal reconstruction of *Rhamphorhynchus* in dorsal view with fully extended wings (based on the superb “Darkwing” specimen with preserved wing membrane, after [32]) we aligned the wing elements as line-objects in gliding-position. Distance between the longitudinal axis of the body and the humerus was measured in articulated *Rhamphorhynchus* specimens, and scaled up to calculate the width of the pectoral region of each investigated specimen. Thereafter, the total distance between the wingtips, i.e. wingspan in the reconstructed wing spar was measured (Figure S1). This measurement technique gave a minimal value for the wingspan, because it was solely based on bone-to-bone contact, not considering the distances between the articulated bones in *in-vivo* joints. Since BSPG 1960 I 470a and IPB 179 had no wing elements preserved, we generated a dataset on femoral length and estimated wingspan of 17 *Rhamphorhynchus* specimens (see Table S1), and fitted a regression line to these data points. The obtained regression equation revealed the relation between femur length and wingspan, and enabled us to estimate the wingspan of the incomplete BSPG 1960 I 470a and IPB 179, as well.

Body masses were calculated based on the equation of Witton [33] given for basal pterosaurs:
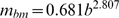
where *m_bm_* is body mass (kg) and *b* is wingspan (m). Although the authors are aware of Witton's [33] comment on *Rhamphorhynchus* being atypically lightweight for its wingspan (i.e. applying this equation gave almost two times heavier body mass for *Rhamphorhynchus* than that based on their skeletal modeling), the absolute body mass values in themselves were not relevant in the current study. The critical parameters in evaluating the potential life history strategies were those potential percentage values of adult body size at which there is a distinct change in the histological pattern. The modeling and mass estimation of the skeleton of each studied specimen *sensu* Witton [33] is well beyond the scope of this paper.

## Results

### Histological description

The terminology used in these histological descriptions follows that of Francillon-Vieillot et al. [34]. Descriptions are presented for each specimen in ascending order of their femoral length. Despite the bad morphological and macrostructural preservation of some specimens and the microcracks that mesh most of the sectioned bones, it can generally be said that the histological details of all specimens are clearly discernible.

### BSPG 1960 I 470a

#### Tibia (Figure 1A)

**Figure 1:**
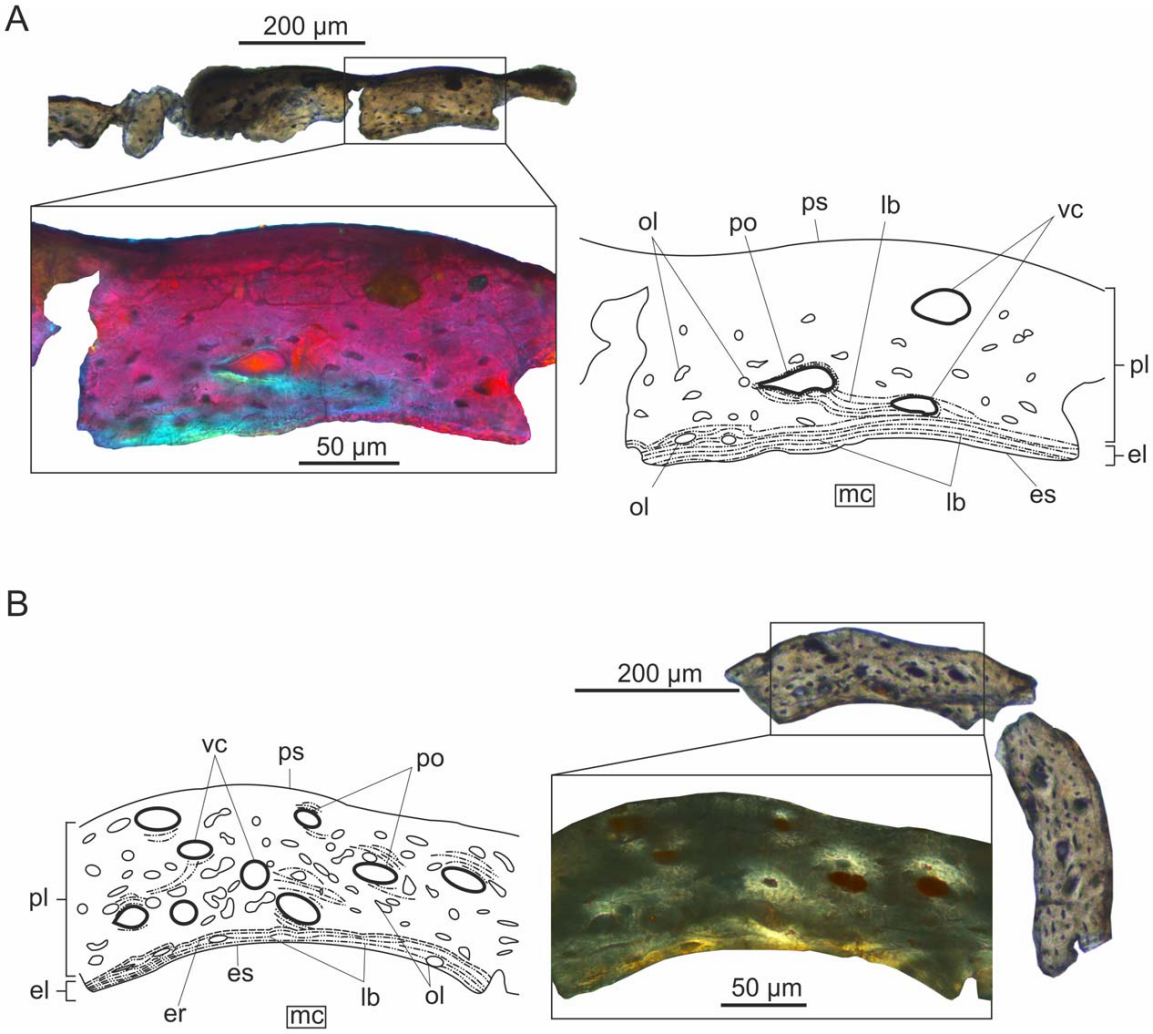
Transverse histological sections under crossed polarizers, and corresponding interpretative drawings of early juvenile long bones of *Rhamphorhynchus muensteri*. (A) The cortex of the tibia in the smallest studied specimen BSPG 1960 I 470a is dominated by immature fibrolamellar bone. It is noteworthy that already in this early ontogenetic stage endosteal bone rims the medullar cavity. (B) The femoral cortex of BSPG 1877 X I reveals more mature fibrolamellar bone with numerous longitudinal vascular canals resulting in high degree of porosity. Both specimens exhibit large, plump osteocyte lacunae.

A thin layer of lamellar bone of endosteal origin rims the medullar cavity. Although only a small piece of the cross section of the tibia can be observed in the thin section, there seem to be only a few longitudinally oriented vascular canals, but these have rather large diameter in relation to the overall thickness of the cortex (Pp = 4.1%). The bone matrix is typically woven with some poorly defined, immature primary osteons, hence the majority of the cortex does not show the mature fibrolamellar pattern yet. The osteocyte lacunae are large and plump throughout the cortex, and possess an extremely well-developed system of dense, radially oriented canaliculi implying extensive communication and nutrient-exchange between the osteocytes. No LAGs or any other growth marks can be observed.

### BSPG 1877 X 1

#### Femur (Figure 1B)

Endosteal coating formed by lamellar bone is deposited on the inner surface of the cortex, the majority of which consists of well-vascularized fibrolamellar bone (Pp = 6%). Embedded in the woven tissue of periosteal origin mature primary osteons are laid down centripetally around the longitudinally oriented and wide open vascular canals. The osteocyte lacunae are large throughout the compacta, but rather elongated in the lamellar, and plump in the woven component of the bone matrix. Dense network of canaliculi is present around the cell lacunae. Sharpey's fibres running obliquely to the outer surface of the femur and crossing almost the entire cortex can also be observed. No LAGs or any other growth marks are present.

### IPB 179

#### Femur (Figure 2A)

**Figure 2:**
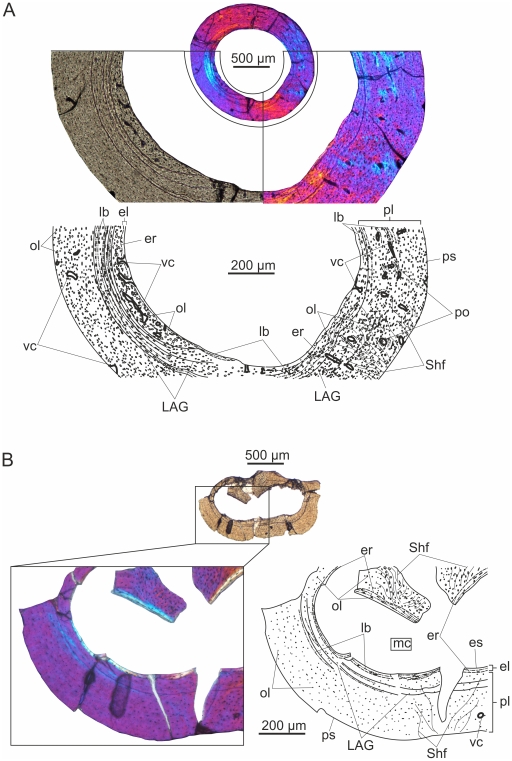
Histological sections of long bones of IPB 179 viewed with polarized microscopy. (A) The femoral cortex shows three periosteal layers of different tissue types. Only a small portion of the oldest, innermost parallel-fibred layer with high vascularization degree is retained. The middle layer is a thick annulus formed of circumferential lamellae and numerous LAGs. The outer periosteal layer is parallel-fibred with moderately large osteocyte lacunae arranged in circular rows. The vascularity is locally different and some vascular canals open onto the periosteal surface. (B) Almost avascular, woven bone with a few deep intracortical LAGs makes up the bulk of the cortex of the tibia which is locally invaded by Sharpey's fibres.

The large gap spanning between the relative body size of this and the smaller specimens is also reflected in the overall histology of the femur which hardly resembles that found in BSPG 1877 X 1. Apart from the consistent, but very thin endosteal coating of the medullary cavity, the majority of the cortex is formed of primary parallel-fibred and not fibrolamellar bone. The total porosity percentage of this section of the femur is Pp = 3.5%.

The deposited tissues and the degree of vascularity are not uniform throughout the periosteal compacta. The oldest periosteal tissue in the deepest cortex is mostly eroded by medullary cavity expansion and deposition of endosteal bone. However, a significant amount is preserved on the left side of the section. It has a parallel-fibred matrix, is more densely vascularized with wide open channels organized in an irregular pattern (Pp_l_ = 10.8%; Pp_t_ = 13%), and has larger, plumper osteocyte lacunae compared to the majority of the cortex.. Endosteal bone was deposited around some blood vessels connecting the medullary cavity and primary bone, thus some of the vascular canals cross the endosteal lamellae and connect to the medullary cavity. This small, primary cortical area is sandwiched between the endosteal bone and a ring of circumferentially oriented lamellae with at least three LAGs, sparse, longitudinal vascular canals and numerous large, flattened osteocyte lacunae. The latter, lamellar layer with the described characteristics is termed an annulus. The overall expansion of the medullary cavity thus destroyed most of the earlier deposited parallel-fibred, densely vascularized tissue, and its drift resulted in the asymmetrical loss of a large portion of the annulus as well.

Peripheral to the annulus, the periosteal cortex is parallel-fibred with numerous, mostly moderately large osteocyte lacunae that are arranged more or less in circular rows. However locally, where the cortex is invaded by Sharpey's fibres, larger, plump and irregularly arranged cell lacunae also occur. Based on the degree of vascularization (Pp_l_ = 3.2%; Pp_t_ = 87%) two distinct areas can be sequestered in the outer compacta. Peripheral to the region where the thicker portion of the lamellar ring borders the earlier periosteal bone, the parallel-fibred cortex contains only a few longitudinal vascular canals. In contrast, the rest of this area is well-vascularized with mostly longitudinal and a few anastomosing vascular canals organized in primary osteons. Several vascular canals open onto the outer surface of the cortex suggesting that the bones of the animal were still capable of further diametrical growth at the time of death.

#### Tibia (Figure 2B)

Distinct but rather thin endosteal lamellae with large, elongate osteocyte lacunae rim the medullary cavity, in which no trabeculae can be observed. Peripheral to the endosteal layer the entire periosteal cortex consists of almost avascular (Pp = 0.24%) woven bone that is locally invaded by dense bundles of Sharpey's fibres. In these areas the osteocyte lacunae are large and plump, whereas in the majority of the primary cortex they are small, round and sparse. The few, longitudinal and narrow vascular canals are organized in immature primary osteons defining an especially poorly vascularized fibrolamellar complex. At least two LAGs can be identified in the deep cortex. There is some lamellar bone deposited on the peripheral side of the LAGs, however, this pattern is still not as distinct as to refer to it as an annulus. No EFS can be observed.

### MTM V 2008.33.1

#### Ulna (Figure 3)

**Figure 3:**
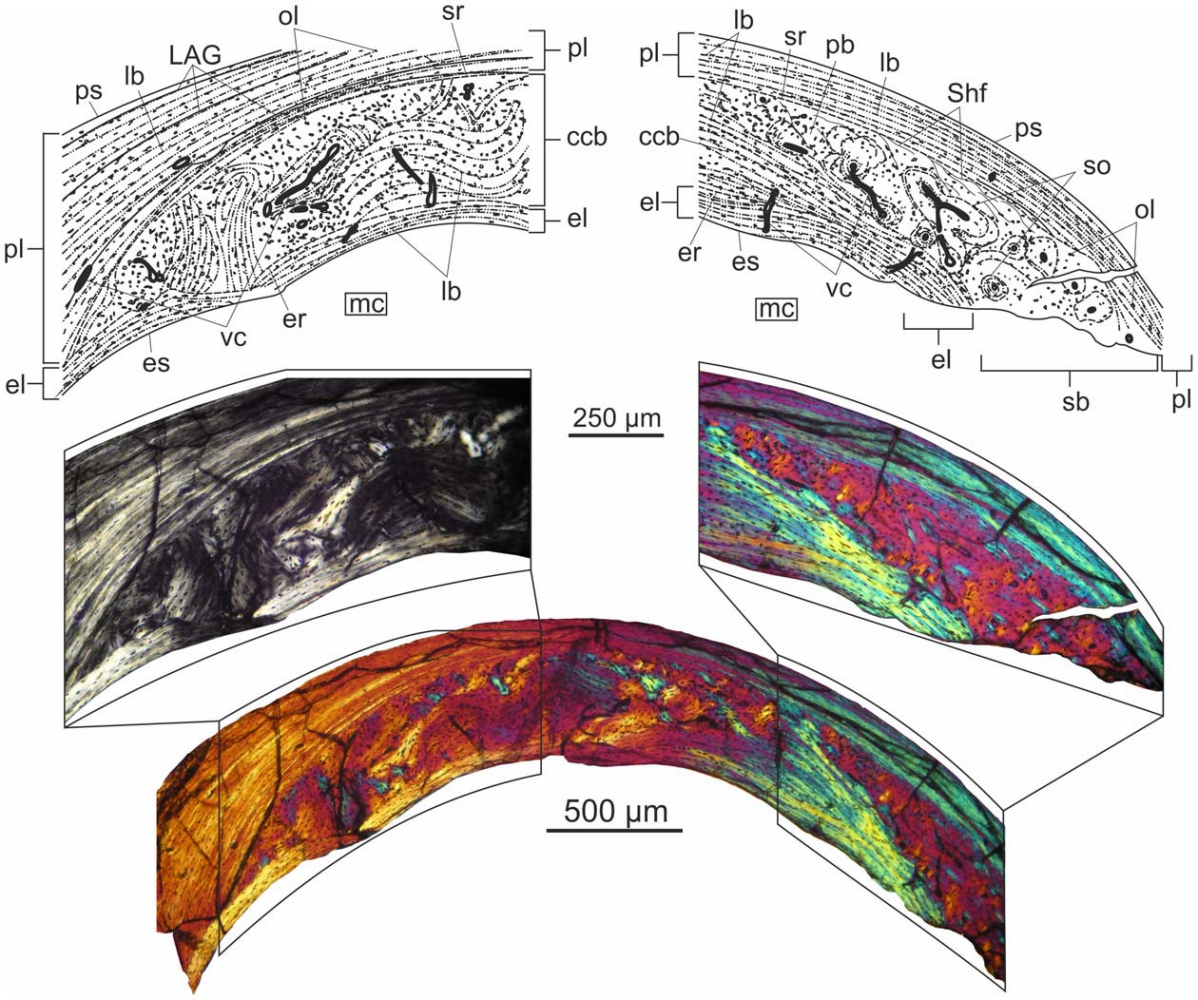
Ulnar histology of MTM 2008.33.1. under crossed polarizers. Peripheral to the thick endosteal layer, extensive compacted coarse cancellous bone forms the middle layer of the cortex. Locally, the middle cortical layer consists of loose Haversian bone. The outer periosteal layer is composed of a strongly asymmetrical OCL.

Thick endosteal circumferential lamellae rim the innermost cortex. No remnants of trabeculae in the medullary cavity can be distinguished.

The inner layer of the periosteal cortex has been remodeled by secondary bone tissue with longitudinal or irregularly anastomosing pattern of vascularization (Pp_l_ = 3.6%; Pp_t_ = 91.7%). Some vascular canals are also seen in the endosteal circumferential lamellae or may open into the medullary cavity. In about one third of the entire cross section of the ulna, the secondary osteons of the remodeled layer are embedded in a mesh of strongly anisotropic and interwoven bundles of lamellae that run irrespectively of the orientation of vascular canals and do not participate in the formation of the osteons. This “whirly” pattern corresponds to that seen in compacted coarse cancellous bone, where cancellous bone is converted into compacted coarse bone due to endosteal infilling of intertrabecular spaces with lamellar bone [34], [35], and indicates that this area was once located close to the metaphyseal region at an earlier ontogenetic stage [35]. Compacted coarse cancellous bone has been described in the wing bones of other pterosaurs, as well [7], but not in the ulna of RAM V97017/258. Besides compacted coarse bone, the remainder of the remodeled layer consists of loose Haversian bone with secondary osteons, and with small patches of primary bone still distinguishable. This small primary area is composed of bundles of longitudinally running fibres, and probably implies the presence of muscle tendons incorporated into the deeper cortical tissue. In a lot of cases the circumferential lamellar organization in the secondary bone is very weak around the secondary osteons. In these cases there is only a thin layer of concentric lamellae adjacent to the resorption line, but mostly woven or longitudinally running fibres in the area between the resorption line and the central vascular canal, which thus appears almost entirely isotropic under crossed nicols. This type of secondary osteons has been identified in the Haversian bone of other pterosaurs, too [7]. Secondary osteons are organized around longitudinal as well as oblique, circular and radial vascular canals. The relative abundance and diverse orientation of secondary osteons throughout the remodeled periosteal cortex differ from those found in other pterosaurs with generally clustered pattern or low number of secondary osteons which are oriented longitudinally in the wing bones [7]. The osteocyte lacunae in this cortical layer are generally large, and their orientation follows that of the fibres in the matrix, accordingly they appear plump or more elongate. In some regions Sharpey's fibres invade the cortex.

The primary periosteal outer layer consists of circumferentially oriented lamellae (OCL) with low vascularity degree (Pp_l_ = 0.5%; Pp_t_ = 8.3%) and at least three LAGs. In some regions the lamellae of the OCL diverge, and distinct thin zones of woven to parallel-fibred bone and annuli of lamellar bone can be observed. There are some peculiarities in the appearance of the OCL, on the basis of which it is differentiated here from the typical external fundamental system (EFS). For instance, at the left side of the section the OCL becomes progressively thicker eventually occupying the majority of the cortex in this area, and finally reaching the endosteal bone with which it converges. The border between the converging endosteal and periosteal lamellae is marked by a clear resorption line. In this region, the periosteal lamellae are perforated mainly by scattered longitudinal and anastomosing vascular canals, and have large, flattened osteocyte lacunae, whereas the rest of OCL is almost avascular with much smaller osteocyte lacunae. The notably asymmetrical constitution of OCL implies strong lateral cortical drift in the sampled region of the ulna. Furthermore this microstructure indicates slowly accreted bone in the peripheral region of the compacta.

The total porosity percentage of this section of the ulna is Pp = 2.4%.

#### III. phalanx of fourth manual digit (wingfinger) (Figure 4)

**Figure 4:**
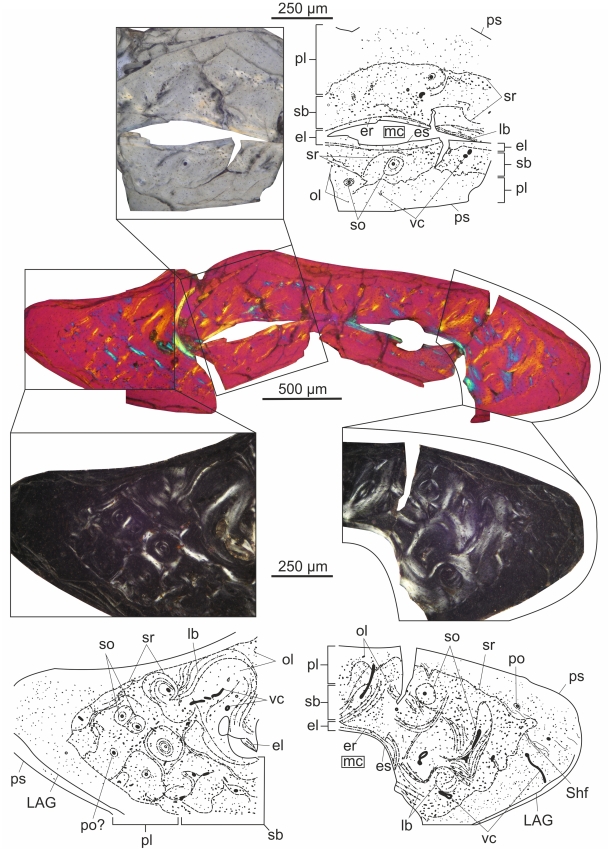
Histology of the III. phalanx of the wingfinger in MTM 2008.33.1. under normal polarized light and crossed nicols. The medullary cavity is lined by a narrow layer of endosteal bone. The middle cortical layer is composed of loose Haversian bone. The outermost, primary layer is formed of sparsely vascularized fibrolamellar bone with one peripheral LAG.

**The** medullar cavity of the third element of the distal wing spar is lined by a moderately thick layer of secondary endosteal bone with flattened osteocyte lacunae. No trabeculae are present in the medullary cavity.

The next layer consists of secondary loose Haversian bone with a matrix of mainly woven and less lamellar component, longitudinal and anastomosing vascularization, and wide open canals (Pp_l_ = 2.2%; Pp_t_ = 93.1%). The density of vascular canals is locally different with the anterior and posterior edges of the wing phalanx being the most densely vascularized, whereas other areas are perforated by more scattered vascular canals. The osteocyte lacunae are large, but rather elongate in the lamellar and plump in the woven component of the bone matrix. Similar to the condition found in the loose Haversian bone of the ulna, the secondary osteons can be characterized by underdeveloped lamellar organization, and have longitudinal as well as transversal orientations. The center of some secondary osteons contains a smaller secondary osteon of a second generation.

The outermost, periosteal layer of the cortex is as thick as the secondary middle layer. It consists entirely of woven bone matrix but with low vascularity degree (Pp_l_ = 0.3%; Pp_t_ = 6.9%) and fewer and generally smaller osteocyte lacunae. The few longitudinal vascular canals are organized in primary osteons forming a very simple type of fibrolamellar bone. The border between this primary outer and the secondary middle layer of the cortex is marked by an irregular, distinct resorption line. Only a faint LAG and no true EFS can be observed in the most peripheral region of the outer cortex.

The total porosity percentage of this section of the III. wingfinger phalanx is Pp = 1.5%.

#### Femur (Figure 5)

**Figure 5:**
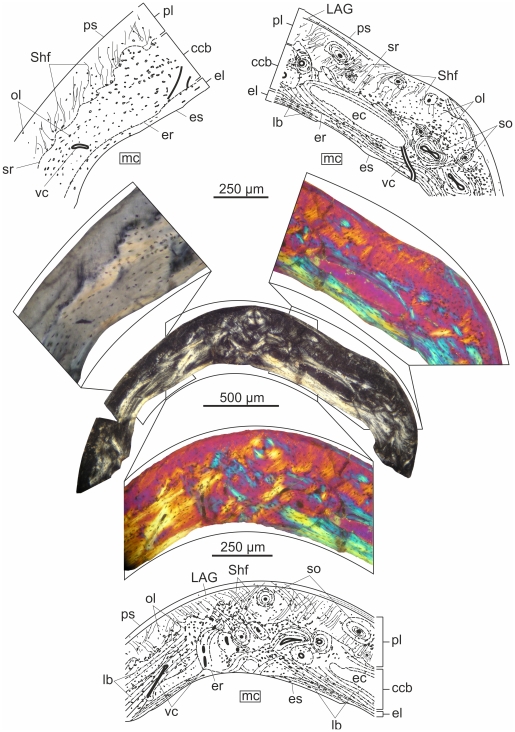
Femoral histology of MTM 2008.33.1. under normal polarized light and crossed nicols. Thick endosteal layer merges into compacted coarse cancellous bone which forms the majority of the cortex. The periosteal outer layer is parallel-fibred, almost avascular with a few scattered secondary osteons and dense network of Sharpey's fibres.

The osteohistology of this element shows clear evidence of strong secondary remodeling in the majority of the cortex, and is similar in some respects to the ulna, in others to the wingfinger phalanx.

The perimedullary endosteal bone is thick with large, flattened osteocyte lacunae. Similarly to the condition found in the ulna, the next layer is composed of compacted coarse cancellous bone with secondary osteons of different maturity. Most secondary osteons are mature and show the characteristic extinction pattern under crossed nicols, whereas a few exhibit secondary osteons of a second generation like those found in the ulna and III. wingphalanx. The vascular canals of the compacted coarse layer are of intermediate lumen and organized in a longitudinal to reticular pattern (Pp_l_ = 2.6%; Pp_t_ = 88.9%). The osteocyte lacunae are numerous, large, and elongate or plump according to fiber orientation. A large erosion room appears adjacent and parallel to the endosteal circumferential layer, and is surrounded by highly organized lamellae. This structure may be derived from erosion processes initiated by medullary expansion.

The outermost, primary layer of the cortex is formed regionally of woven but mostly parallel-fibred periosteal bone that is extensively invaded by a dense network of Sharpey's fibres. Similar to the structural separation of primary and secondary tissues in the wingphalanx, the border between the compacted coarse cancellous bone and the periosteal layer is marked by a scalloped resorption line. On either side of this cement line the osteocyte lacunae are large and plump, whereas in the more peripheral region of the periosteal cortex they are small and sparse. A few longitudinally oriented and well-developed secondary osteons are scattered in the periosteal layer. Peripheral to the most distinct secondary osteon in the section, there is a small area exhibiting bundles of longitudinally running fibres also observed in the ulna. Apart from the longitudinal vascular canals provided by the sporadic secondary osteons (Pp_l_ = 0.4%; Pp_t_ = 11.1%) the periosteal cortex is avascular. A single LAG runs in the outermost region but there is no change in tissue type deposited along this growth line, thus no true EFS can be observed.

The total porosity percentage of this section of the femur is Pp = 1.6%.

#### Tibia and fibula

These two elements of the lower leg are fused at the level of the section, and both exhibit the same histological features as the tibia of IPB 179. Although the diameter of the tibia is 2–2.5 times that of the fibula, the distinct endosteal lamellar layer with large, elongate osteocyte lacunae is as thick or locally thicker in the fibula than it is in the tibia. No trabeculae can be observed in the medullary cavity.

Peripheral to the endosteal layer the entire cortex is composed of almost avascular periosteal woven bone that is locally invaded by Sharpey's fibres. The osteocyte lacunae are small, round and sparse. There are only one or two, longitudinally oriented vascular canals in the periosteal cortex that are organized in primary osteons. However, secondary osteons of wide lumina are lined up at the fusion zone, which most probably represented the main and common supply channel for both elements (Pp = 0.3%). The circular lamellae of these secondary osteons interweave with the endosteal lamellae that line the medullary cavities of the tibia and fibula. In both elements there are four LAGs in the periosteal cortex, three of which run in the middle region so close to each other that two of them converge locally. The most peripheral LAG runs close to the outer surface of the compacta. No distinct layer of lamellar bone is deposited adjacent to the LAGs, thus no zones, annuli or EFS can be distinguished.

#### Prepubis

The microstructure of this accessory element of the pelvis is similar to that of the tibia and fibula. The medullary cavity is divided into two main chambers that are separated by the thick endosteal circumferential lamellae surrounding each chamber. The secondary endosteal layer has large, elongate osteocyte lacunae. No trabeculae in the medullary cavity are present.

The periosteal cortex consists mainly of woven bone with few, longitudinal vascular canals that are organized in primary osteons. Lateral to the medullary chambers, two large secondary osteons invade the primary cortex. The primary and secondary osteons together give a relatively high porosity percentage for the entire cortex (Pp = 2.7%). A very distinct and straight bundle of Sharpey's fibres runs perpendicular to the bone surface, crosses the entire periosteal cortex closing in the nearby secondary osteon, and reaches down into the endosteal lamellae. The osteocyte lacunae are generally small, round and sparse throughout the periosteal cortex, however, they are significantly larger and plump in the region invaded by Sharpey's fibres. Two LAGs can be identified one of which is positioned in the middle, the other in the outer part of the cortex. In a restricted portion of the section, there is a local increase in the amount of lamellar component peripheral to the outer LAG, but no definite annulus or EFS can be observed.

### BSPG 1929 I 69

#### Femur (Figure 6A)

**Figure 6:**
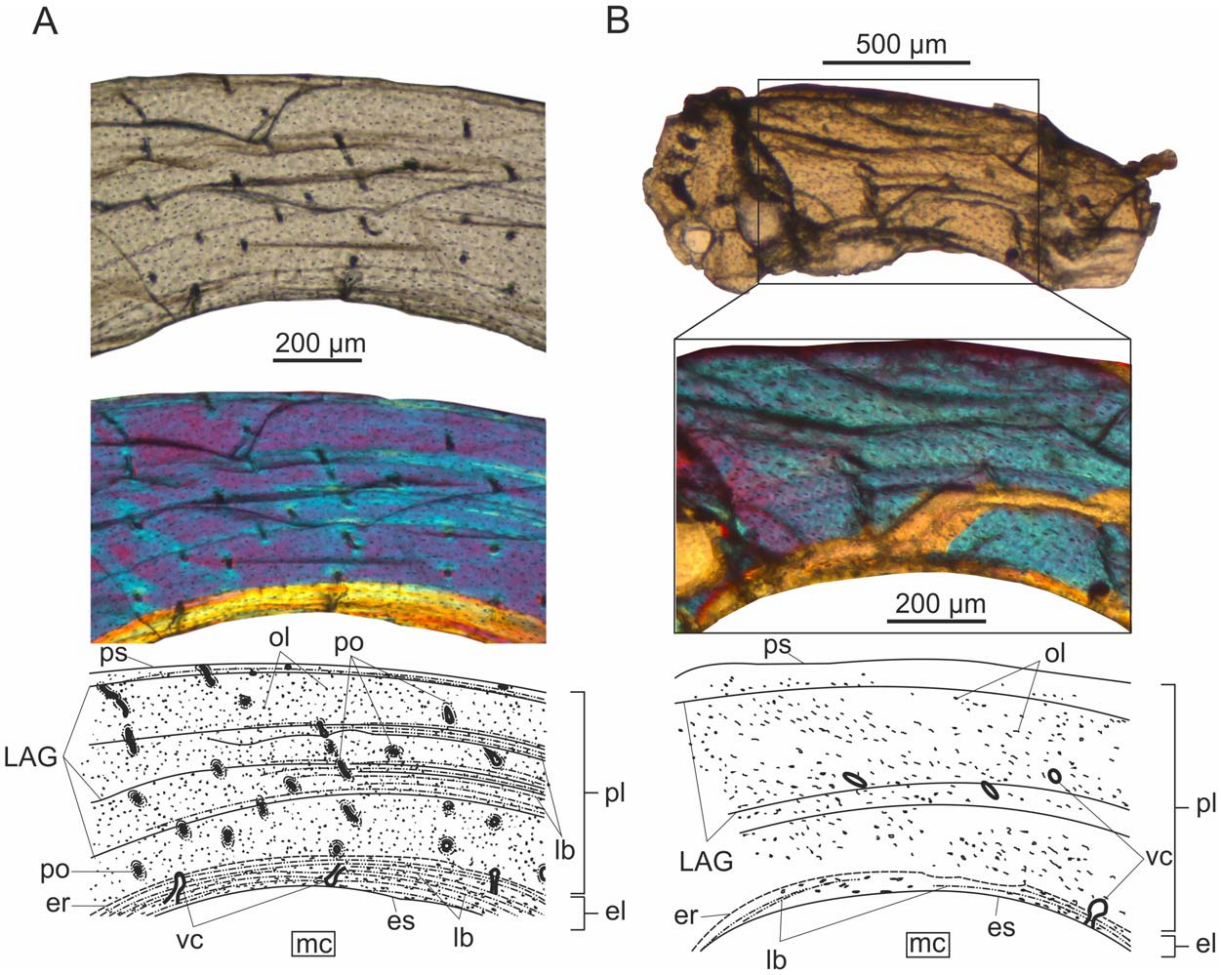
Histological sections of long bones of BSPG 1929 I 69 viewed with polarized microscopy. (A) The femoral cortex has a very thick layer of endosteal lamellae. The parallel-fibred periosteal bone is moderately well vascularized by mostly longitudinal channels. Deep intracortical LAGs within three to four thin, lamellar-fibred annuli are present. (B) The medullar cavity of the tibia is lined by endosteal bone. The bulk of its cortex is composed of an almost avascular, parallel-fibred primary bone tissue with a few, widely spaced LAGs.

Similarly to IPB 179, this femur has gone through more growth cycles with intermediate depositional rate interrupted by dramatic decrease or cessation of appositional growth.

A strongly anisotropic, thick layer of secondary endosteal bone with circumferentially oriented lamellae and distinct resorption line is present in the innermost cortex. A few radial and oblique vascular canals cross these lamellae and open onto the surface of the medullary cavity. No trabecular struts can be observed. The small osteocyte lacunae are more elongate or flattened in the endosteal lamellae than in other regions of the cortex where they are rather round.

The bulk of the cortex is composed of primary, parallel-fibred bone with several, mostly longitudinally oriented and homogeneously dispersed vascular canals of intermediate diameter (Pp = 2.4%). A number of oblique, rather short anastomoses can also be observed. Thus, the vascular canals surrounded by primary osteons have mostly longitudinal spatial organization throughout the cortex. No fibrolamellar bone tissue is retained in this section of the femur. Five to six LAGs within three to four thin, lamellar-fibred annuli are present. The first annulus is found in the innermost quarter of the cortex thickness and contains one LAG. The next annulus runs approximately in the cortical midline with two, very closely spaced LAGs. The following annulus also contains two, closely spaced LAGs, which converge in other parts of the section. The distance between these three annuli slightly varies; they converge in some part of the cortex and spread in the other. The most peripheral annulus with one LAG lies very close to the preserved outer surface of the bone. In the regions of annuli there is no apparent change in the pattern or degree of vascularization. Locally, Sharpey's fibres cross the periosteal cortex. No secondary osteons or any other evidence of Haversian remodeling are present, nor is there an EFS.

#### Tibia (Figure 6B)

This section is rather distorted by a large fracture, but a small piece of the fibula can still be recognized attached to one corner of the sectioned part of the tibia. A distinct but rather thin layer of lamellar endosteal bone lines the medullary cavity.

The entire primary periosteal cortex is almost uniformly composed of parallel-fibred bone tissue with true lamellar component present only in the primary osteons, and a scant lamellar layer lines one or two LAGs. The cortex is poorly vascularized with longitudinal vascular canals of small as well as large diameter (Pp = 1.9%). Primary osteons surrounding the narrow vascular canals are rather immature, whereas those organized around the wide canals are more developed. The presence of a single secondary osteon visible at one corner of the section implies that this area was close to the fusion zone of the tibia and fibula. The small osteocyte lacunae are round or somewhat flattened throughout the periosteal cortex and oriented parallel to the fibres of the matrix. Owing to the dominance of parallel-fibred tissue in the periosteal cortex no distinct annuli can be observed. In spite of this four to six LAGs in four, relatively widely spaced groups can still be distinguished. However, due to the severe diagenetic distortions, the exact number of LAGs cannot be defined. No traces of Haversian remodeling or an EFS can be observed.

#### Size-classes vs. ontogenetic stage

Based on the length of humerus and first wing-phalanx, BSPG 1877 X I represents the lowest value of Bennett's [3] first, smallest size category, whereas MTM V 2008.33.1. and BSPG 1929 I 69 can be assigned to the second (medium) and third (largest) size-class, respectively. Estimated lengths of wing elements extrapolated from femoral length rates IPB 179 in the lowest range of the medium size-class, whereas BSPG 1960 I 470a falls below the registered smallest size category of Bennett [3].

Based on the histological features of BSPG 1960 I 470a and BSPG 1877 X I, these specimens are graded as early juveniles. On the same basis, IPB 179 represents a late juvenile-subadult specimen, whereas MTM V 2008.33.1. and BSPG 1929 I 69 both reveal adult histology in their bones.

#### Wingspan and body mass estimates

**Figure 7:**
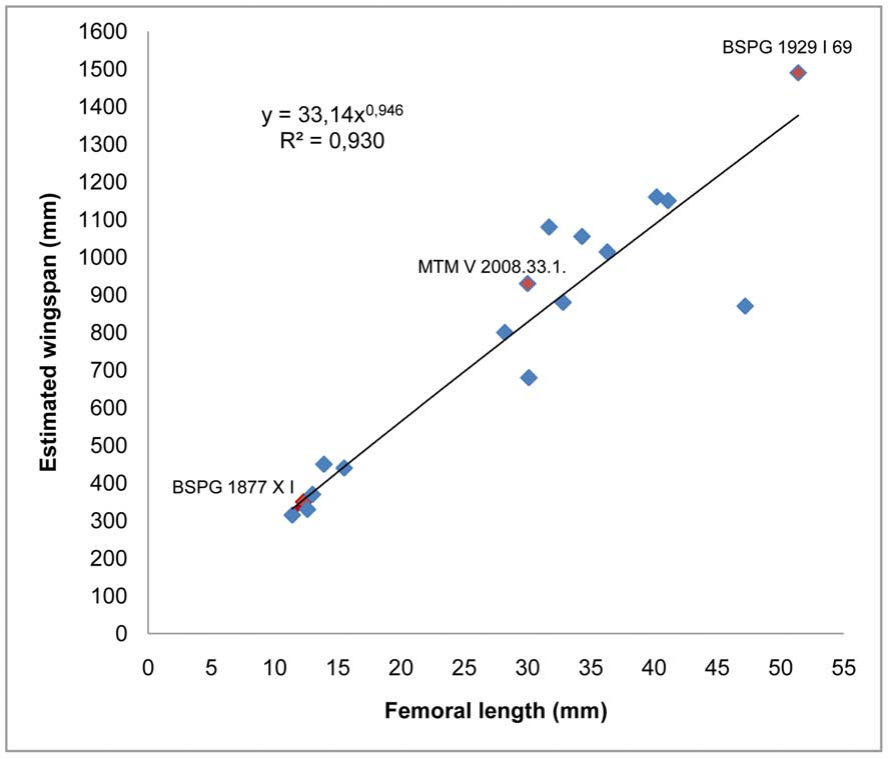
Plot and regression of femoral length vs. estimated wingspan of 17 *Rhamphorhynchus* specimens. Changes in the relation between these two traits during ontogeny can best be described by a power function in which high r^2^ value (p = 0.000) guarantees the reliability of the equation making wingspan estimations of specimens with missing wing bones possible.

With most of the wing elements preserved, the wingspan of BSPG 1877 X I, BSPG 1929 I 69, and MTM V 2008.33.1. could be estimated directly from the wing bones resulting in 330 mm, 1490 mm, and 930 mm, respectively (Figure S2). Since there is a strong correlation between femur length and wingspan (r^2^ = 0.9307, p = 0.000), the wingspan of BSPG 1960 I 470a and IPB 179, lacking any of the wing elements, was extrapolated from the regression equation (Figure 7)
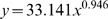
where *y* is wingspan (mm) and *x* is femur length (mm). This equation, which demonstrates how the relation between femur length and wingspan changes during ontogeny, gave the 300 mm and 600 mm wingspan for BSPG 1960 I 470a and IPB 179, respectively (Figure S2). Body masses gained from the equation of Witton [33] along with the estimated wingspan values are given in Table 2.

**Table 2:** Estimated wingspan and body mass of the five investigated specimens.

Specimen	Estimated wingspan (mm)	Estimated body mass (g)
BSPG 1960 I 470a	300*	23
BSPG 1877 X I	330	30
IPB 179	690*	241
MTM V 2008.33.1	930	555
BSPG 1929 I 69	1490	2085

Asterisk indicates that wingspan was deduced from the regression equation describing the relationship between femur length and wingspan.

## Discussion

Ideally, histological studies of an ontogenetic series, which enable reconstruction of developmental processes, should include samples of the same skeletal elements at the same relative location in each studied specimen. Unfortunately, in the current study this requirement was only in part possible to meet. Direct comparison is thus limited to a restricted number of cases, however, some peculiarities as well as a general overview concerning growth strategy can still be assessed based on the overall histological pattern found in the specimens of the ontogenetic series of *Rhamphorhynchus*.

### Comparison of bone tissues within size-categories

The estimated body sizes of BSPG 1877 X 1 and MTM V 2008.33.1. (current study) correspond to those of CM 11433 and RAM V97017/258 [13], respectively. This makes it possible to highlight the histological similarities and differences between specimens of the same size category.

Since there was no common sampled element in BSPG 1877 X 1 and CM 11433 representing the first size-class of Bennett [3] with CM 11433 being slightly larger, only the overall histological pattern found in the bones of these two specimens can be compared. The most important feature is the presence of well-vascularized fibrolamellar bone in the bones of both specimens. Although not all of the sampled bones of CM 11433 have been described in detail [13], the bulk of the cortex in most of the studied long bones consists of densely vascularized woven bone with primary osteons. This equals the condition found in the femoral periosteal cortex of BSPG 1877 X 1. It is noteworthy that in both specimens there is a thin endosteal coating on the inner surface of the cortex, the formation of which is thought to refer to the termination of medullary expansion [11]. The endosteal bone would thus be expected in more advanced ontogenetic stages in pterosaurs with very thin bone walls. However, this tissue type is present even in the smallest investigated specimen (BSPG 1960 I 470a), the body size of which is below the smallest defined size category. The deposition of endosteal bone has not been found in such early ontogenetic stages of other pterosaurs like azhdarchids, pteranodontids [7] or *Pterodaustro* [11]. Endosteal bone formation can be a response to increased or altered stress on the considered element [36], thus the early activation of the endosteum in *Rhamphorhynchus* might be attributed to mechanical requisition already taking effect during early development.

More elements can be compared in MTM V 2008.33.1. and RAM V97017/258 which both match the second size-class of Bennett [3]. Apart from sexual dimorphism, possible factors responsible for the observed histological differences are discussed separately for each sample.

The microstructure of the ulna differs in many respects in MTM V 2008.33.1. and RAM V97017/258. These discrepancies probably reflect the different sampling location in the two cases which was more proximal in MTM V 2008.33.1. and mid-shaft in RAM V97017/258. Secondary bone tissue prevails in the inner cortex of the ulna in MTM V 2008.33.1., but there is no trace of secondary remodeling in the ulna of RAM V97017/258. The periosteal cortex of RAM V97017/258 is entirely composed of parallel-fibred bone with decreasing peripheral vascularity. The middle layer of compacted coarse cancellous bone and loose Haversian bone, and the outer periosteal lamellar layer characterizing the ulna of MTM V 2008.33.1. are all absent in the ulna of RAM V97017/258. The overall histological pattern suggests that, in contrast to RAM V97017/258, the sampled region of the ulna of MTM V 2008.33.1. once belonged to the metaphysis that had been subjected to considerable structural rearrangement by cortical and medullary cavity drift. It is very likely that the extensive remodeling processes and this pattern of drift, which is so unusual in the case of bones with roughly circular cross section, also reflect the strong mechanical requisition related to the flight stroke, in which this bone region was exposed to high deflection and torsional loads [7], [37], [38]. Based on the histological comparison of the ulnae it is unclear which specimen ranks higher in ontogenetic maturity.

The elements of the wingfinger of RAM V97017/25 and MTM V 2008.33.1., though not the same phalanges in the two specimens, show more similarities than the ulnae. The differences that might be attributed to the different position of second and third phalanx in the wing spar, however, are still considerable. Although the structural division of the cortex into three distinct layers can be regarded as a common feature, the middle and outer layer is composed of different tissue types in the two specimens. Whereas the middle cortical layer in MTM V 2008.33.1. is composed of secondary loose Haversian bone, the same layer consists only of periosteal fibrolamellar bone in RAM V97017/258. The outer cortex is primary and almost avascular in both specimens, however, with woven bone matrix in MTM V 2008.33.1. and parallel-fibred in RAM V97017/258. This makes it difficult to interpret which specimen can be considered more mature. The secondary remodeling refers to a more advanced ontogenetic stage of MTM V 2008.33.1. than that of RAM V97017/258 with purely primary cortical bone. However, the depositional rate of woven bone with irregularly running fibres revealed in the outer cortex of the third wingphalanx of MTM V 2008.33.1. is higher than that of spatially more organized parallel-fibred bone described in the second wingphalanx of RAM V97017/258. Higher depositional rates are expected in earlier ontogenetic stages, hence implying the more mature state of RAM V97017/258. However, given the equal body size of these specimens it is unlikely that later during ontogeny of MTM V 2008.33.1. the woven peripheral bone tissue would have all been removed, and thereafter the periosteum would have formed a novel, entirely parallel-fibred cortex. The higher number of preserved LAGs in RAM V97017/258 cannot be used as indicator of a more mature ontogenetic stage either, since secondary remodeling in MTM V 2008.33.1. could have potentially removed former intracortical growth lines. Hence, similar to the ulnae, the histology of the phalanges does not clarify the relative ontogenetic order of these specimens.

The femora also reveal extensive biases in the two specimens. Compacted coarse cancellous bone of secondary origin dominates the inner, and woven to parallel-fibred periosteal bone the outer half of the femoral cortex in MTM V 2008.33.1. In RAM V97017/258 the cortex peripheral to the endosteal layer is uniformly composed of parallel-fibred primary tissue. For the same reason pointed out in the comparison of the wingphalanges, the number of LAGs does not necessarily indicate a more mature ontogenetic state of RAM V97017/258 over MTM V 2008.33.1. Unlike the sectioned mid-diaphyseal region of the femur in RAM V97017/258, the sampled region of the femur of MTM V 2008.33.1., must have formed once the metaphysis of the growing bone. Similar to the ulnae, the different femoral sampling locations in the two specimens most likely account for the described histological differences. The femoral microstructure distorted by secondary processes in MTM V 2008.33.1. does not allow the ontogenetic ordering of these specimens relative to each other.

The histology of the tibiae of RAM V97017/258 and MTM V 2008.33.1. is more similar than that of any other compared elements. Peripheral to the endosteal layer, the entire cortex of the tibia of both specimens consists of almost avascular primary bone and contains a number of LAGs. However, this periosteal tissue is composed of woven bone with primary osteons (fibrolamellar *sensu lato*) in MTM V 2008.33.1., whilst entirely parallel-fibred bone in RAM V97017/258. This might refer to a more mature ontogenetic status of RAM V97017/258 over MTM V 2008.33.1., however, it would be difficult to see how this transition in the deposited bone could have happened in the two, equally large specimens.

The histological comparison of the elements of MTM V 2008.33.1. and RAM V97017/258 revealed major differences that cannot all be attributed to the differences in sampling locations (e.g. discrepancies in the peripheral tissue types of the wing phalanges, or those in the periosteal bone tissues of the tibiae), but rather speak for intraspecific variability, maybe sexual dimorphism.

### Assessment of ontogenetic ages based on size vs. histology

The ontogenetic validity of the smallest size category of Bennett [3] is clearly supported by the overall microstructure found in the bones of the three small specimens BSPG 1960 I 470a, BSPG 1877 X I, CM 11433. The histological features of the cortex of the tibia in BSPG 1960 I 470a meet the expectations based on the overall body size of the specimen by showing unambiguously its early juvenile or status. However, all cancellous bone typical for embryonic skeletal elements has been resorbed in this specimen, which confirms that it was not an embryo. The overall histology of the femur of BSPG 1877 X 1 strongly resembles that of the tibia of BSPG 1960 I 470a. However, BSPG 1877 X 1 clearly represents a more mature stage of ontogeny, which is manifested by the exhibition of well-developed primary osteons. The microstructural features of the investigated elements of BSPG 1960 I 470a, BSPG 1877 X 1 and CM 11433 confirm the juvenile nature of all three specimens. The finer histological details such as the maturity state of primary osteons or the morphology of osteocyte lacunae also correspond to the relative size differences between these individuals.

The ontogenetic composition of the second size-class is more complex. Histological comparison of IPB 179, RAM V97017/258 and MTM V 2008.33.1. reveals many significant differences even allowing for the sampling biases in MTM V 2008.33.1. Based on their similar body sizes, RAM V97017/258 and MTM V 2008.33.1. were expected to show similar histological traits, i.e. to represent about the same developmental state. Still, the overall histological pattern described in both the femur and tibia of RAM V97017/258 rather equals to that found in BSPG 1929 I 69, even though the latter is assigned to the largest (adult) and not the medium (subadult) size category of Bennett [3]. Thus the microstructure of the femur and tibia definitely ranks RAM V97017/258 and BSPG 1929 I 69 at the same, adult ontogenetic stage in the series. On the basis of the tibia, which is the only directly comparable element between IPB 179 and MTM V 2008.33.1., these two specimens would represent the same ontogenetic stage despite their different estimated body sizes. On the other hand, other histological features retained in the peripheral periosteal cortex of the femur of MTM V 2008.33.1. clearly suggest that this specimen was more mature than IPB 179. In contrast to IPB 179, which exhibits a number of vascular canals opening onto the outer surface of the femur and has larger, circumferentially organized osteocyte lacunae throughout the femoral cortex, MTM V 2008.33.1. reveals an avascular outermost cortex with smaller, sparse and scattered osteocyte lacunae. This observation is also in accordance with the estimated smaller size of IPB 179. Morphologically MTM V 2008.33.1. is a fully grown, skeletally mature individual [39] which is also supported by the histological features of its bones, whereas the bone microstructure of IPB 179 still shows clear signs of active growth. Accordingly MTM V 2008.33.1. as well as RAM V97017/258 can be considered adults, whereas IPB 179 should be referred to as late juvenile or subadult at best. In sum, IPB 179, RAM V97017/258 and MTM V 2008.33.1. do not represent the same ontogenetic stage despite that all belong to the second size-class of Bennett [3]. Hence, the second size category includes subadult as well as adult individuals, thereby disproving the pure subadult composition of this size-class suggested by Bennett [3].

Bennett's [3] assumption that the third size class is composed of adult individuals is supported by the histological features of the only specimen that matches this size range. However, based on the situation found in MTM V 2008.33.1. and RAM V97017/258, adult size can be considerably smaller than the defined lowest limit of the third size-class. Thus the border between the second and third size classes established by Bennett [3] does not bear any real ontogenetic meaning.

### Growth dynamics and life history strategy

The prevalence of well-vascularized, fast growing fibrous to fibrolamellar bone found in the bone cortex of considerably young but certainly not embryonic individuals clearly implies that *Rhamphorhynchus* experienced rapid growth in early ontogenetic ages. The large, plump osteocyte lacunae with extensive network of canaliculi are also indicative of high metabolic rate in the bone tissues of these early juveniles. Fibrolamellar bone with the same characteristics has also been observed in the radius and second wing phalanx of CM 11433 [13]. However, this bone tissue is the simplest form of the fibrolamellar complex in the manner of vascularization pattern with few but widely open, almost exclusively longitudinally oriented vascular canals. Vascularity is also relatively low when compared to the laminar, plexiform or reticular fibrolamellar bone found in most dinosaurs (e.g. sauropods), but comparable to the vascularization degree seen in the primary bone of smaller maniraptorans like *Velociraptor*, *Shuvuuia* or *Confuciusornis* [29] which are believed to have had growth rates well within the endotherm range [29], [40]. The largely longitudinal vascularization and relatively low porosity in the early juveniles of *Rhamphorhynchus* may suggest an overall slower growing tissue compared to other defined fibrolamellar tissue types. However, all forms of fibrolamellar bone have considerably higher growth rates than do any kind of lamellar-zonal bone [13], [41]. Thus, early juvenile growth rate in *Rhamphorhynchus* exceeded the general level found in extant non-avian sauropsids. Considering the demonstrated variability in adult body size with estimated wing spans and body masses ranging from 930 mm and 555 g in MTM V 2008.33.1 to 1490 mm and 2085 g in BSPG 1929 I 69, the body size of the investigated early juveniles can be given as minimum and maximum percentages of adult body size. BSPG 1960 I 470a and BSPG 1877 X 1 exhibit 20–32% and 22–35% of adult wingspan and 1–4% and 1.5–5.4% of adult body mass, respectively. These values also refer to the very early ontogenetic status of these specimens.

As development proceeds, changes in the counteracting periosteal and endosteal activity determine the overall microstructure of growing bones. On one hand, the deposited periosteal bone tissue alters from fast growing fibrolamellar to mainly slower growing parallel-fibred bone organized in superimposed zones with sparse vascularization and deep intracortical growth lines. On the other hand, the majority of fibrolamellar bone in the deeper cortex vanishes due to the extensive secondary remodeling and resorption processes associated with medullary expansion and cortical drift. The rate of these changes cannot be determined precisely; however, based on the degree of vascularization, the formation of secondary tissues (loose Haversian bone, compacted coarse cancellous bone) must have been relatively fast. Traces of fibrolamellar bone *sensu lato* can still be observed in most elements of MTM V 2008.33.1., and also in the tibia of IPB 179, but none in BSPG 1929 I 69. Nonetheless, the relative timing of the deceleration of appositional bone growth and the onset of secondary remodeling is hard to estimate. Owing to the large ontogenetic gap between the smaller and the larger specimens in which the secondary resorption and remodeling processes have largely removed or obscured the inner, older periosteal bone, a distinct zone of transition could not be pointed out. IPB 179 only possesses 50–75% of adult wingspan and 15–40% of adult body mass, and has not retained fibrolamellar bone in its femoral cortex. This indicates that the deposition of slow growing parallel-fibred bone took over relatively early during ontogeny. Thus this alteration must have happened when the animal had a wingspan and body mass somewhere between 30–50% and 7–20% of that of the adults, respectively. Whether the fast growth phase had sudden or rather gradual termination cannot be determined on the basis of the specimens investigated in this study. However, an abrupt change from the deposition of fibrolamellar bone in the inner cortex to the formation of parallel-fibred bone in the outer cortex has been reported in the wing phalanx of RAM V97017/258 [13].

The presence of LAGs in the woven bone of some elements, and thin, widely spaced annuli with lamellar bone and one or more LAGs between zones of parallel-fibred bone in others indicates periodic slow-down and cessation of appositional growth during the slower growth phase. However, there is no sign of an EFS in any of the adult specimens. The formation of an EFS refers to the plateau in the growth curve indicating that the animal has reached asymptotic body size [35], [42]–[44]. Consequently the presence of an EFS is generally thought to be associated with determinate growth strategy *sensu stricto* in which growth ceases after the organism reaches its maximum body size [45]. If not a preparational or preservational artefact, the absence of an EFS along with the observed histological structures most probably means that even the largest studied specimen BSPG 1929 I 69 was still capable of further residual growth when it died. If *Rhamphorhynchus* had determinate growth strategy *sensu* Lincoln et al. [45] or determinate growth type I *sensu* Sebens (genetically determined asymptotic adult size with little modification possible [46]), the lack of an EFS most probably implies that this individual had not reached its final body size by the time of its death (but see the absence of an EFS in some ratites, [47]), despite other histological features which indicate dramatic slow-down of growth. Alternatively, *Rhamphorhynchus* might have had indeterminate growth *sensu* Lincoln et al. [45] or determinate growth type III *sensu* Sebens (attenuating growth with the absence of a known achieved asymptote [46]). In this case growth trajectories can be very variable under different environmental circumstances but after a certain incipient growth period only limited changes are possible [46]. This growth strategy characterizes most extant non-avian sauropsids [48]–[52] with the exception of some turtles [53].

In general, determinate growth strategy (type I) among amniotes can be characterized by the dominance of fibrolamellar bone in the early ontogenetic stages and by the formation of an EFS at the residual growth stage. When animals with determinate growth approach their maximal body size, the deposition of fibrolamellar or parallel-fibred bone tissue (fast and moderately fast growing, respectively) is succeeded by the formation of outer circumferential lamellae, indicating a rather abrupt termination of somatic growth. In contrast, indeterminate growth strategy (or determinate growth type III) is associated with the deposition of slow-growing lamellar-zonal bone in which the zones of active growth consist mainly of parallel-fibred bone in early juveniles (but fibrolamellar bone may also be present, [54], [55]) and of lamellar bone interrupted by tightly spaced LAGs in more mature animals (cf. [56]). Indeterminate growth dynamics can be characterized by a relatively low growth rate in juveniles and a gradual slowing down of growth until the animal dies.

Based on bone microstructure, *Rhamphorhynchus* seems to show a mixture of characteristics of the ‘typical’ indeterminate and determinate growth strategy (*sensu* [45]). Growth dynamics that can be characterized by two distinct phases, a fast growing early juvenile phase and a slower growing late juvenile-subadult phase is rather typical of determinate growth strategy. On the other hand, the alternating deposition of the thick zones of parallel-fibred bone typically found in extant non-avian sauropsids, and the thin, lamellar-fibred annuli with LAGs but no EFS in more mature specimens refers to the phases of active bone growth with intermediate rate that were slowed down or interrupted from time to time, but did not come to a definite termination. This pattern clearly speaks for indeterminate growth strategy. Nevertheless, there is no sign of increasing density of lamellar component toward the outer cortex or of typical lamellar-zonal cycles (like e.g. in crurotarsi or “pseudosuchians”, [54]). Another possibility to consider is that *Rhamphorhynchus* had determinate growth but with a certain degree of developmental plasticity. Developmental plasticity could have involved differential growth rates in corresponding ontogenetic stages of different *Rhamphorhynchus* individuals which could have resulted in a significant variability in final body sizes. This would mean that some could attain fairly large body sizes, whereas others grew substantially smaller depending on individual genetic background and/or environmental factors in their life histories, similarly to some extant vertebrates (cf. [18]) or the extinct *Plateosaurus* [44]. This hypothesis is not only supported by the substantial difference in body size between the two adult specimens MTM V 2008.33.1. and BSPG 1929 I 69, but also by the histological discrepancies revealed in the similar-sized MTM V 2008.33.1. and RAM V97017/258. High variance in final body size seems to apply to other pterosaur groups [10], e.g. the pterodactyloid ornithocheirids too, and probably accounts for a great deal of debate on their taxonomy (e.g. [57]–[60]). In ornithocheirids some specimens of the same species seem to be skeletally mature but significantly smaller than others with still immature, growing skeletons (E.P. pers. obs.). Although no statistical or detailed comparative morphological analysis has been conducted on this subject yet, this phenomenon might refer to developmental plasticity in ornithocheirids. The low sample size of the current study prevents us to examine whether size- and histological differences correlate and show a bimodal distribution in *Rhamphorhynchus* which would imply that these differences lie in sexual dimorphism. In any case, much more histological sampling would be needed from individuals of the medium and largest size category to test these hypotheses by revealing whether the lack of an EFS is the normal condition in the adults of *Rhamphorhynchus*, and if not, whether distinct size categories could be assigned to the appearance of an EFS.

Albeit the ontogenetic series of this study is incomplete, the overall histological pattern evident in *Rhamphorhynchus* is equivalent to that found in the ctenochasmatid *Pterodaustro* [11] and in some respects strongly resembles that reported in enantiornithine birds such as *Concornis* [28] or *Gobipteryx* [27], and the primitive bird (or unenlagiine dromaeosaurid *sensu* [61]) *Rahonavis* [27]. Surprisingly enough, this obvious similarity in the overall pattern of bone histology described in *Pterodaustro* and enantiornithines has apparently remained undetected, because the transition from fibrolamellar bone to superimposed zones of parallel-fibred bone in the cortex during ontogeny has been explained by quite different life histories. In *Pterodaustro* it has been interpreted as the sign of attaining sexual maturity which has re-allocated the energy from growth to reproduction causing a definite slow-down in bone-growth [11]. The same phenomenon in enantiornithines has been explained by the early onset of flight; a capability comparable to superprecocial birds, such as some extant megapod species that are able to take off immediately after hatching. In the latter case, the energy distribution among different life history components during ontogeny was not re-organized by reproduction but powered flight [27].

Clearly, the most parsimonious approach is to presume that one and the same factor is responsible for the equivalent histological patterns seen in the two, phylogenetically so distant but in their lifestyle so similar groups. Reconsidering the two suggested triggers, the onset of reproduction may well be the potential explanation for the sharp change in the overall histology during ontogeny of *Pterodaustro*, it has a very weak explanatory value in enantiornithines where the fibrolamellar complex probably exists only during early ontogeny [27]. The deposition of parallel-fibred bone starts already in early juveniles, hence making sexual maturation as the cause of such a change unlikely in these birds. In contrast, the other suggested factor, the onset of flight would adequately explain the observed analogous histological pattern in both groups. Here we prefer the hypothesis that bone growth is slowed down by the initiation of a new, and much more energy consuming locomotory activity, namely powered flight. This decrease in growth rate is marked by the distinct change in the deposition from fast to slow growing bone tissue in *Rhamphorhynchus* as well as in all volant groups (including *Pterodaustro*) that exhibit similar histological pattern. The authors are not aware of any extant flying vertebrates which can reproduce prior to the onset of powered flight. The absence of the latter life history strategy among volant vertebrates is reasonable, since powered flight becomes essential much earlier for the individual's own survival than reproduction does for the long-term survival of its genes.

It follows from this hypothesis that *Rhamphorhynchus* hatchlings grew rapidly and did not take off for steady flight until they reached a minimum size and probably musculoskeletal maturity as well. This is in contrast with the hypothesis of superprecocial flaplings that could fly off immediately after hatching [10], [12]. In fact, the finding of Lü et al. [12] that the ratio of egg mass to adult mass points to low parental investment in *Darwinopterus* in itself contradicts their superprecocial hypothesis. Superprecocial embryos require substantial amount of nutrients stored in their eggs to reach an advanced level of somatic maturity state by the time the embryo hatches [62]. If the egg volume of *Darwinopterus* was relatively as low as that of squamates [12], then how could it have contained so much yolk as to cover the energy requirements of an extremely well-developed, volant hatchling? Furthermore, the prevalence of fibrolamellar bone in the cortex of most wing elements in CM 11433 [13] makes it unlikely that the wing bones were already functional at this post-hatching developmental state [21]. Thus, both the low parental investment reported in *Darwinopterus* [12] and the histological attributes revealed in *Rhamphorhynchus* ([13], and current study) clearly contrast the notion of superprecocial flaplings. *Rhamphorhynchus* was indeed an early flier when compared to most extant volant vertebrates, but apparently not as superprecocial as some megapod birds are today.

According to the hypothesis presented here, the onset of powered-flight in *Pterodaustro* occurred after attaining 53% of adult size (based on the length of different skeletal elements, [11]), whereas this change in locomotory activity in *Rhamphorhynchus*, based on our samples, might have occurred much earlier, approximately at 30–50% of adult wingspan or 7–20% of adult body mass. However, owing to the inadequate sample size, the question remains whether there was a definite critical body size at which *Rhamphorhynchus* began to fly similarly to *Pterodaustro*, or the timing of the first take off mainly depended on individual variance in life history parameters. Nevertheless, the rapid growth of the non-volant hatchlings may suggest parental care that extended for a short period and could have involved feeding and/or protection of the neonates until they were mature enough to fly off and take care of themselves. Alternatively, *Rhamphorhynchus* hatchlings could have been precocial to the effect that they could have left their nests immediately after hatching, but they must have been exclusively terrestrial or rather arboreal. They could have clambered around quadrupedally on the branches of trees feeding themselves with smaller invertebrates or vertebrates without any parental contribution. Gliding from one tree to another or down to the ground is also conceivable; however, the mechanical loads of gliding on the bones and muscles could have required a certain level of somatic maturity, too. The presence of endosteal bone even in the smallest specimens implies a very early but moderate mechanical recourse of the limb bones, which also supports precocial lifestyle for *Rhamphorhynchus*.

After becoming airborne, growth of *Rhamphorhynchus* continued at moderate rates but still significantly increased body size. The early acquisition of parallel-fibred bone in *Rhamphorhynchus* ([13], and current study) is most probably a secondary evolutionary reversal in the pterosaurian lineage, since earlier pterosaurs show higher growth rates at corresponding developmental stages [7], [13], [16], [17]. Hence, it is very likely that *Rhamphorhynchus* was a relatively much earlier flier than more basal or derived pterosaurs with fibrolamellar bone deposition in this ontogenetic stage. Whether growth of *Rhamphorhynchus* was determinate like that of *Pterodaustro* and other derived pterosaurs where the close spacing of growth lines or the presence of an EFS directly suggest the cessation of growth, or indeterminate like in modern non-avian sauropsids, is still dubious. Due to the uncertainties surrounding the applicability of growth mark counts in assessing individual ages [63]–[67], the absolute time necessary to reach a potential asymptotic body size cannot be estimated either. However, the resemblance of overall histological pattern in *Rhamphorhynchus* and *Pterodaustro*, and the presence of an EFS in full-grown specimens of other, more basal pterosaurs such as *Dorygnathus* [16] or *Dimorphodon* [7] imply that it is only a question of further sampling to find the traces of an EFS in the larger specimens of *Rhamphorhynchus* too.

**Figure 8:**
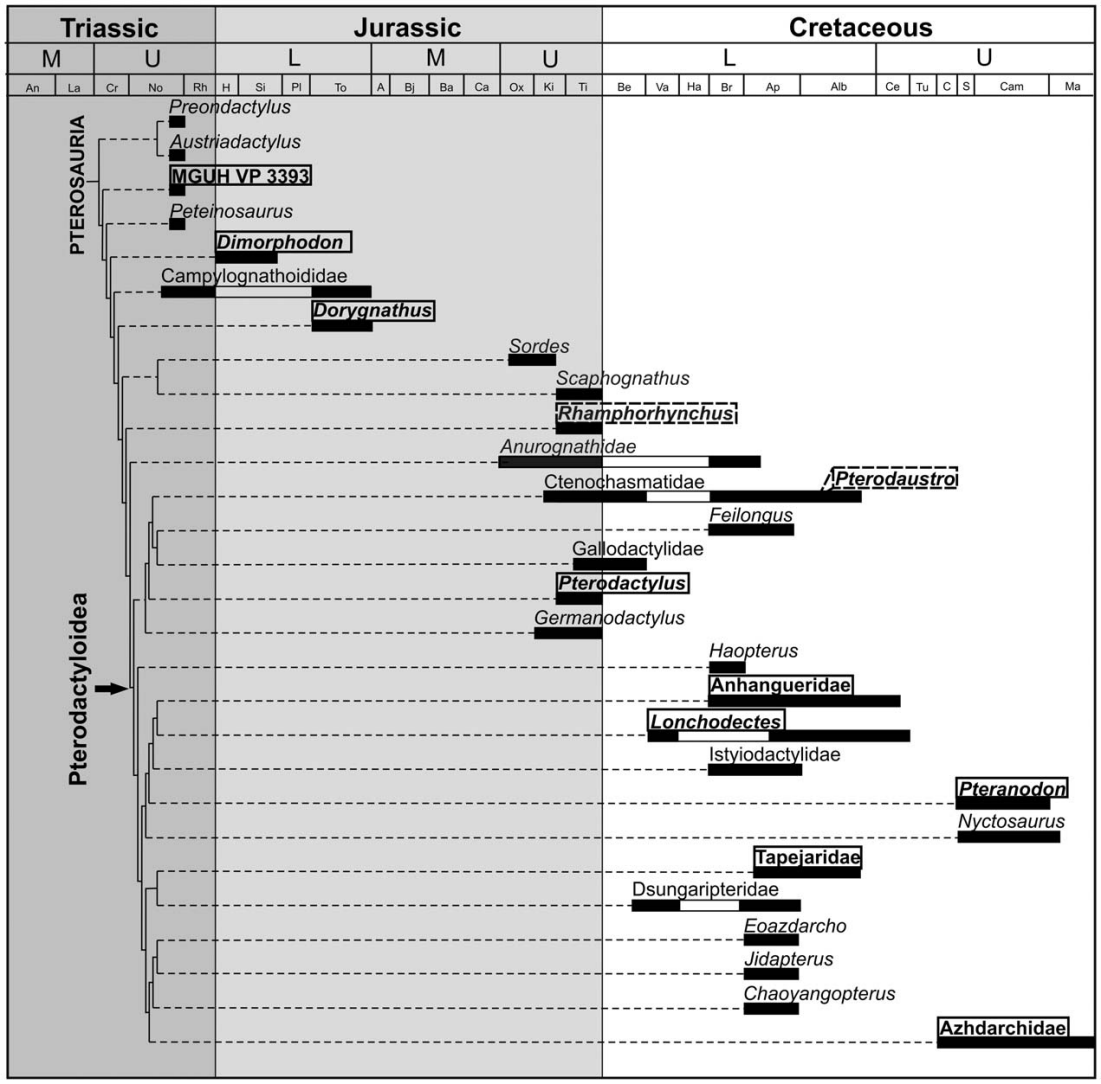
Phylogenetic interrelationships, temporal ranges and general bone histological characters of different members of Pterosauria. Taxa are specified at generic or family level. Taxa that have been histologically investigated so far are framed in rectangles. Those framed by rectangles of solid line have predominantly fibrolamellar bone of varying vascularity degree in the majority of the cortex, occasionally with LAGs and EFS indicating dramatic slowing down of growth in late ontogenetic stages, in which the onset of flight may have occurred. The two genera distinguished by frames of broken lines, *Rhamphorhynchus* and the ctenochasmatid *Pterodaustro* differ from the latter in revealing fibrolamellar bone only in juvenile stages and parallel-fibred bone with LAGs in later stages of development. We hypothesize that the early slowing down of growth in these genera is due to the relatively early onset of powered flight. Phylogenetic interrelationships without implications for divergence times are modified after [15] for non-pterodactyloids, and after [66] for Pterodactyloidea. Temporal ranges and chart are modified after [68]–[70].

In the light of the histological results it becomes evident that there is no universal pattern in the growth strategy of pterosaurs (Figure 8, based on [15], [68]–[70]). In this respect, pterosaurs seem to reveal a degree of complexity that is comparable to that of early birds. Based on the bone microstructure found in Late Triassic and Early Jurassic genera, in which fibrolamellar bone predominates most of the cortex, fast and determinate growth up to adulthood was probably the plesiomorphic state in pterosaur growth dynamics. Although absolute growth rates seem to vary between these taxa too, they all were most likely incapable of flight until approaching or reaching final body size. At least by the Late Jurassic a new strategy appeared, in which fast growth only characterized early juveniles, and with the relatively early onset of flight growth slowed down appreciably but continued for long time. This growth strategy, which we inferred for *Rhamphorhynchus*, might have been determinate or indeterminate. Whether it should be considered as an evolutionary reversal with respect to slow growth during the majority of development or rather an innovation in terms of early flight is debatable. *Pterodaustro* from Early Cretaceous seems to show a similar growth pattern with a relatively early onset of flight, however with a prolonged phase of fast growth compared to that of *Rhamphorhynchus*, and with determinate growth. Other pterosaur taxa from the Early and Late Cretaceous reveal fast and determinate growth, which can be interpreted as retaining or secondarily returning to the plesiomorphic strategy. Thus the apparent diversity in growth strategies among pterosaurs warns us that there is no universal pattern that can be extrapolated to pterosaurs in general.

### Summary

Our investigation shows that, in contrast to Bennett's [3] suggestion, the second size category of *Rhamphorhynchus* does not only include subadult but also adult specimens, hence it cannot be used as an indicator of real ontogenetic stage. Body sizes of histologically adult specimens can vary significantly, which implies developmental plasticity in this respect. Based on microstructural features of the ontogenetic series, *Rhamphorhynchus* had retained a high initial growth rate in hatchlings and early juveniles. After this fast growth phase, at a relatively early stage in ontogeny, growth slowed down remarkably, but continued for a longer period of time. Here we suggest that the factor responsible for the transition from fast to slow growth phase is the onset of powered flight which redirected energy from growth to this expensive locomotory activity. Furthermore we assume that the similar histological pattern found in *Pterodaustro* [11] can also be attributed to the onset of flight and not to the onset of reproduction as suggested by Chinsamy et al. [11]. Our findings also contradict Unwin [10] and Lü et al. [12] by confuting the notion that *Rhamphorhynchus* hatchlings were superprecocial fliers. Our hypothesis is further supported by the low parental investment demonstrated in *Darwinopterus* [12]. Thus, the rapid growing early juveniles of *Rhamphorhynchus* and probably other pterosaurs as well were either attended by their parents until they got airborne or they were hiding, arboreal animals that could take care of themselves by climbing on trees and feeding on smaller invertebrates or vertebrates. After attaining a certain somatic maturity they flew off and lived the same lifestyle as their volant parents. Because of the lack of an EFS in the largest studied specimen, it is still uncertain whether *Rhamphorhynchus* had deterministic growth or not. However its overall histological pattern, and the presence of an EFS in other pterosaurs rather speak for a determinate growth strategy. Histological studies hitherto conducted on a variety of pterosaurs from the entire temporal range of the clade demonstrate that instead of a universal pattern that could be applied to pterosaurs in general, different taxa displayed notably different growth strategies. This confirms the need for further histological investigations to reveal the variety of life history strategies in this peculiar group of flying vertebrates.

## Supporting Information

Figure S1 **Explanatory drawing of the method used for wingspan estimations.** The skeletal reconstruction of the wing spar of *Rhamphorhynchus* with fully extended wings is based on the ‘Darkwing’ specimen (after Prondvai and Hone, 2008).
(TIF)

Figure S2 **Dimensional differences between the investigated** ***Rhamphorhynchus*** **specimens based on the wingspan estimates.** Note that the wingspan of the largest specimen BSPG 1929 I 69 is five times of that of the smallest specimen BSPG 1960 I 470a.
(TIF)

Table S1 **List of 17** ***Rhamphorhynchus*** **specimens used to reveal the relationship between femoral length and estimated wingspan.** Boldface type marks the histologically investigated specimens.
(TIF)

Text S1 **Institutional abbreviations appearing in the inventor numbers of specimens.**
(DOC)

Text S2 **Abbreviations for histological structures used in the figures.**
(DOC)
