# Supplementary figures and images for: Life History of Rhamphorhynchus Inferred from Bone Histology and the Diversity of Pterosaurian Growth Strategies
Source: PLoS One. 2012 Feb 15;7(2):e31392. doi: 10.1371/journal.pone.0031392 (PMC3280310; doi:10.1371/journal.pone.0031392)

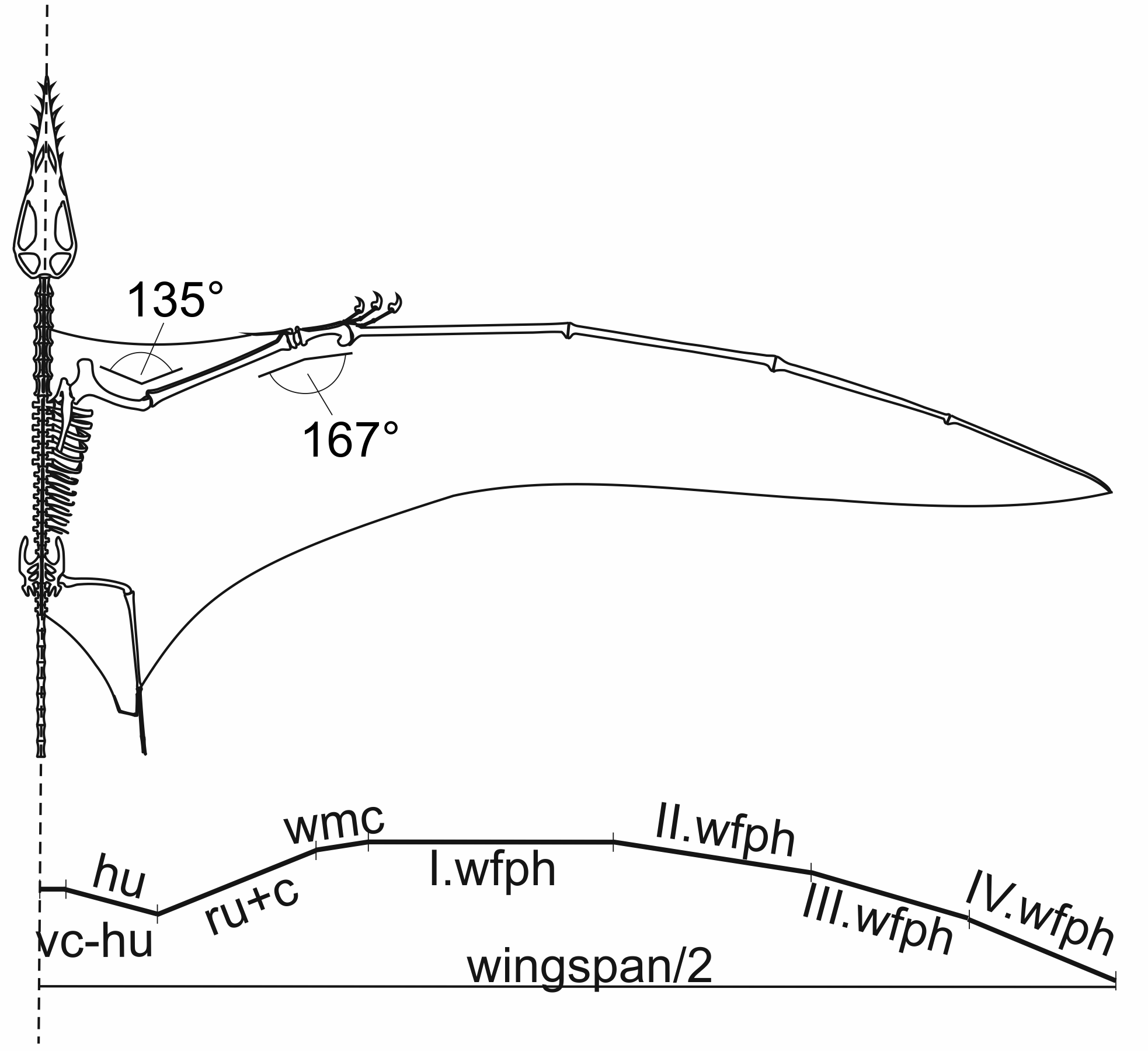

Supplement: Figure S1 — Explanatory drawing of the method used for wingspan estimations. The skeletal reconstruction of the wing spar of Rhamphorhynchus with fully extended wings is based on the ‘Darkwing’ specimen (after Prondvai and Hone, 2008). (TIF) [file pone.0031392.s001.tif]

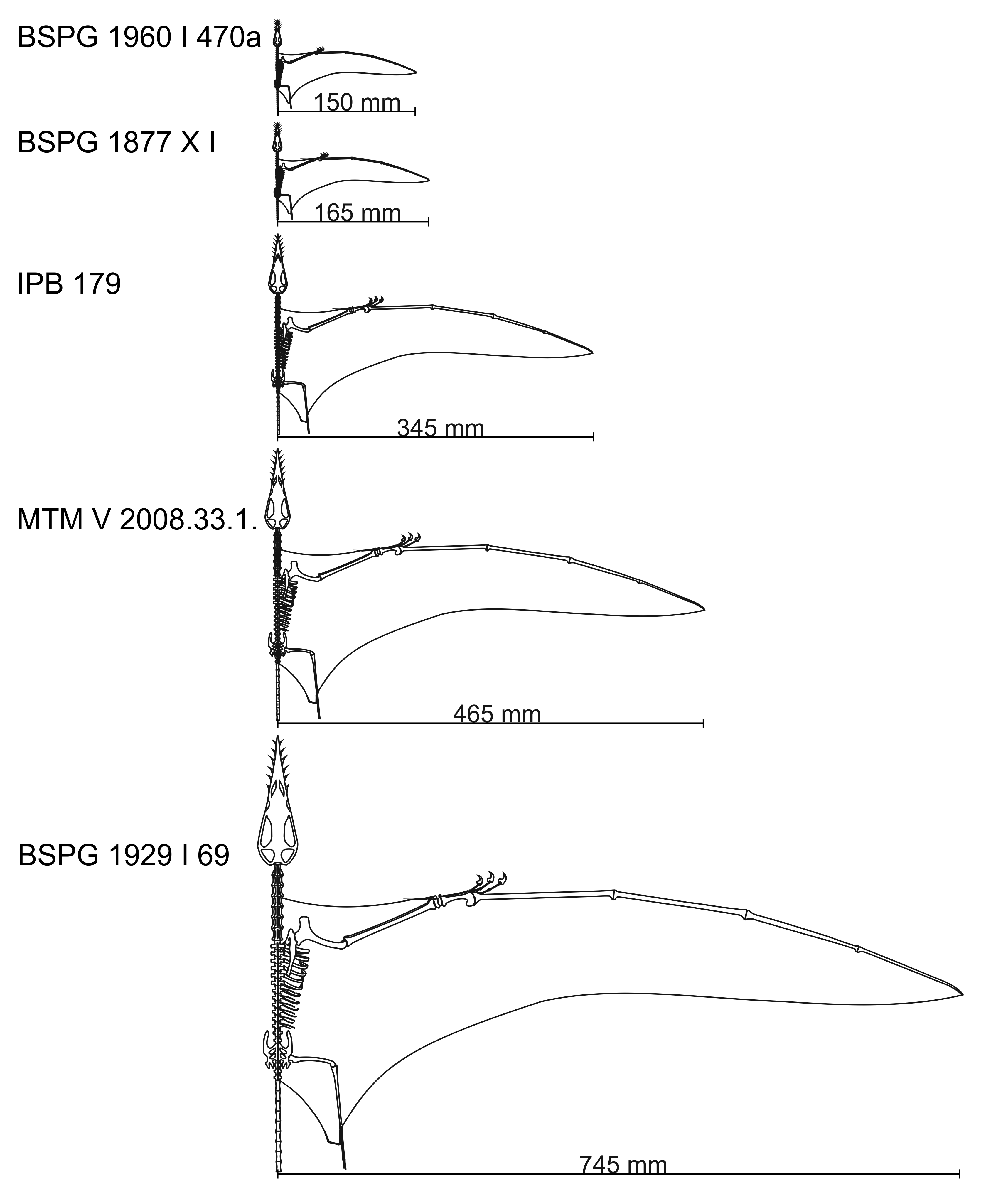

Supplement: Figure S2 — Dimensional differences between the investigated Rhamphorhynchus specimens based on the wingspan estimates. Note that the wingspan of the largest specimen BSPG 1929 I 69 is five times of that of the smallest specimen BSPG 1960 I 470a. (TIF) [file pone.0031392.s002.tif]

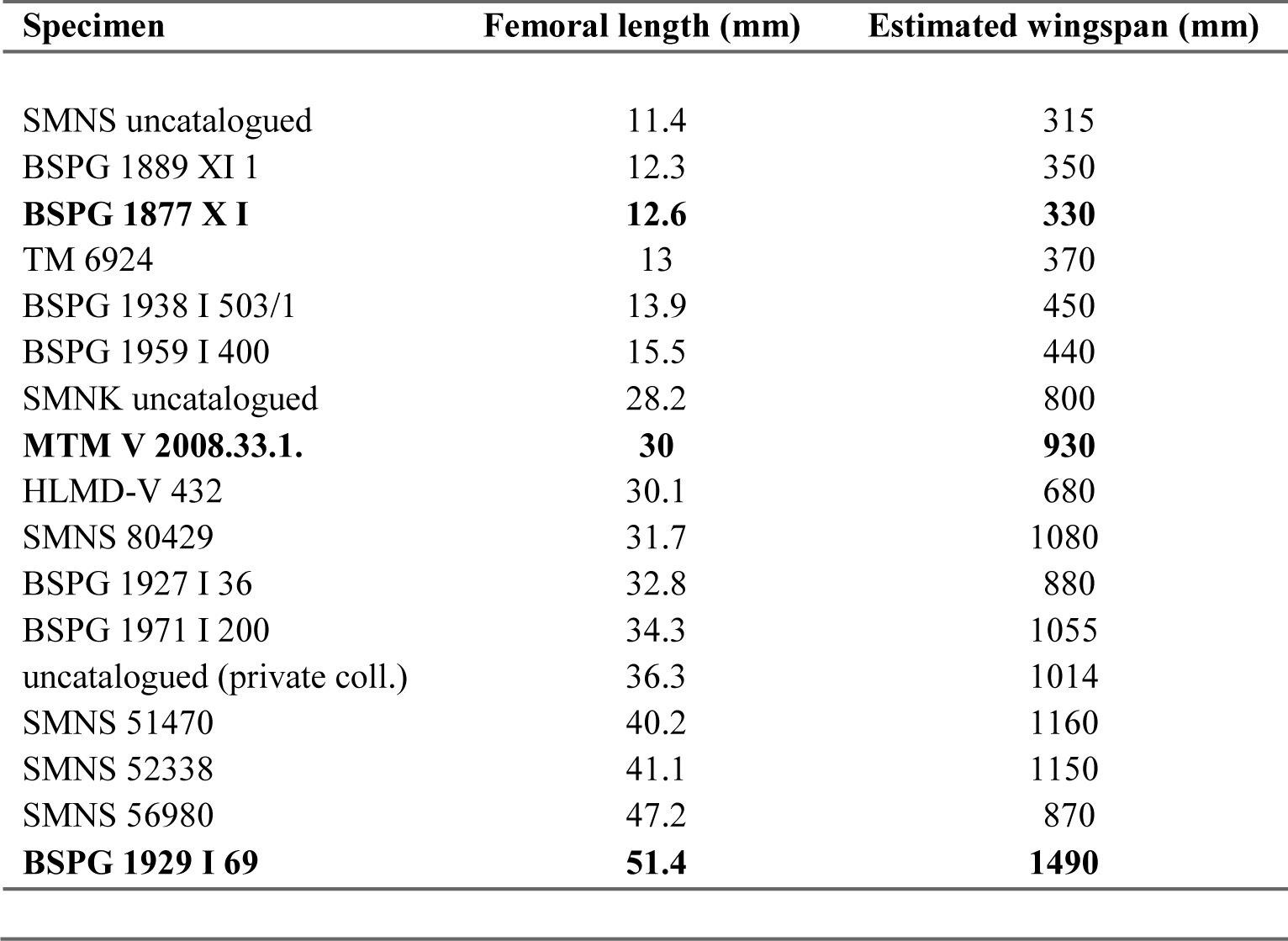

Supplement: Table S1 — List of 17 Rhamphorhynchus specimens used to reveal the relationship between femoral length and estimated wingspan. Boldface type marks the histologically investigated specimens. (TIF) [file pone.0031392.s003.tif]
