# Supplementary material for: Life History of Rhamphorhynchus Inferred from Bone Histology and the Diversity of Pterosaurian Growth Strategies
Source: PLoS One. 2012 Feb 15;7(2):e31392. doi: 10.1371/journal.pone.0031392 (PMC3280310; doi:10.1371/journal.pone.0031392)
Supplement: Text S1 — Institutional abbreviations appearing in the inventor numbers of specimens. (DOC) [file pone.0031392.s004.doc]

**Institutional abbreviations:** BSPG, Bayerische Staatsammlung für Geologie und Paläontologie, München, Germany; CM, Carnegie Museum of Natural History, Pittsburgh, USA; HLMD, Hessisches Landesmuseum, Darmstadt, Germany; IPB, Steinmann Institut für Geologie, Mineralogie und Paläontologie, Bonn, Germany; MGUH, Geological Museum, University of Copenhagen, Denmark; MTM, Hungarian Natural History Museum, Budapest, Hungary; RAM, Raymond Alf Museum, Claremont, USA; SMNS, Staatliches Museum für Naturkunde, Stuttgart, Germany; SMNK, Staatliches Museum für Naturkunde, Karlsruhe, Germany; TM, Teylers Museum, Haarlem, Netherlands
