# Supplementary material for: Life History of Rhamphorhynchus Inferred from Bone Histology and the Diversity of Pterosaurian Growth Strategies
Source: PLoS One. 2012 Feb 15;7(2):e31392. doi: 10.1371/journal.pone.0031392 (PMC3280310; doi:10.1371/journal.pone.0031392)
Supplement: Text S2 — Abbreviations for histological structures used in the figures. (DOC) [file pone.0031392.s005.doc]

**Histological abbreviations in the figures**

**ccb** compacted coarse cancellous bone **pb** primary bone

**ec** erosion cavity **pl** periosteal layer

**el** endosteal layer **po** primary osteon

**er** endosteal resorption line **ps** periosteal surface

**es** endosteal surface **sb** secondary (loose Haversian) bone

**LAG** lines of arrested growth **Shf** Sharpey’s fibres

**lb** lamellar bone **so** secondary osteon

**mc** medullar cavity **sr** secondary resorption line

**ol** osteocyte lacunae **vc** vascular canal
